# Deciphering the HLA-E immunopeptidome with mass spectrometry: an opportunity for universal mRNA vaccines and T-cell-directed immunotherapies

**DOI:** 10.3389/fimmu.2024.1442783

**Published:** 2024-09-05

**Authors:** Maya Weitzen, Mohammad Shahbazy, Saketh Kapoor, Etienne Caron

**Affiliations:** ^1^ Department of Immunobiology, Yale School of Medicine, New Haven, CT, United States; ^2^ Department of Biochemistry and Molecular Biology and Infection and Immunity Program, Biomedicine Discovery Institute, Monash University, Melbourne, VIC, Australia; ^3^ Yale Center for Immuno-Oncology, Yale Center for Systems and Engineering Immunology, Yale Center for Infection and Immunity, Yale School of Medicine, New Haven, CT, United States

**Keywords:** HLA-E peptides, immunopeptidome, mass spectrometry, vaccine design, viral infection, cancer immunotherapy

## Abstract

Advances in immunotherapy rely on targeting novel cell surface antigens, including therapeutically relevant peptide fragments presented by HLA molecules, collectively known as the actionable immunopeptidome. Although the immunopeptidome of classical HLA molecules is extensively studied, exploration of the peptide repertoire presented by non-classical HLA-E remains limited. Growing evidence suggests that HLA-E molecules present pathogen-derived and tumor-associated peptides to CD8^+^ T cells, positioning them as promising targets for universal immunotherapies due to their minimal polymorphism. This mini-review highlights recent developments in mass spectrometry (MS) technologies for profiling the HLA-E immunopeptidome in various diseases. We discuss the unique features of HLA-E, its expression patterns, stability, and the potential for identifying new therapeutic targets. Understanding the broad repertoire of actionable peptides presented by HLA-E can lead to innovative treatments for viral and pathogen infections and cancer, leveraging its monomorphic nature for broad therapeutic efficacy.

## Introduction

The development of checkpoint blockade immunotherapy, CAR T cells, bispecific T-cell antibodies, mRNA-based vaccines, and other T-cell-based immunotherapeutic strategies have emerged in the treatment landscape over the last decade (1–4). Advances in such immunotherapies often rely on identifying actionable T cell targets represented by relatively short peptide fragments presented by HLA class I or class II molecules. It is now established that those peptides presented by HLA molecules are collectively called the immunopeptidome, which can be interrogated using mass spectrometry (MS) technologies (5). Over the last thirty years, the science of investigating the immunopeptidome, i.e. MS-based immunopeptidomics, has been mostly applied to identify and quantify peptides presented by classical HLA molecules: HLA-A, -B, and -C for class I molecules, and HLA-DP, -DQ, -DR for class II molecules (6–10). Those genes are the most polymorphic across the entire human genome, with more than 27,300 alleles for class I and 11,600 alleles for class II (https://www.ebi.ac.uk/ipd/imgt/hla/about/statistics/), making the human immunopeptidome tremendously complex from a population perspective (11–13). In contrast, very little is known about the collection of peptides that can potentially be presented by non-classical HLA-I molecules (HLA-Ib) (14), including HLA-E, which is relatively non-polymorphic with only two known alleles: HLA-E*01:01 and HLA-E*01:03 (15). Thus, the HLA-E immunopeptidome should be much simpler and widely shared across humans.

Historically, HLA-E was first established as a key regulator of innate immunity (16, 17). Its primary function is to present canonical signal peptides derived from degraded HLA-A, -B, and -C proteins to the CD94/NKG2 receptors on NK cells, thereby inhibiting NK cell functions. However, the experimental detection of HLA-E-restricted CD8^+^ T cells within tumors and in infection profiles of cytomegalovirus (CMV), Salmonella enterica serovar Typhimurium, Mycobacterium tuberculosis (Mtb), human immunodeficiency virus (HIV), and Epstein-Barr virus strongly supports the notion that HLA-E also participates in adaptive immune responses (15). It does so by presenting pathogenic and tumor-associated peptides as ligands for the αβTCR (T-cell receptor) of CD8^+^ T cells (15, 18). The emerging role of HLA-E in CD8^+^ T cell activation has recently increased interest in developing universal vaccination and T-cell-based immunotherapy strategies targeting HLA-E-bound viral or tumor-associated epitopes that may be shared across all individuals.

Given the tremendous advances in MS technologies in recent years, revisiting the composition of the peptide repertoire presented by HLA-E in various disease conditions using state-of-the-art MS-immunopeptidomics methods may thus provide valuable information for new therapeutic approaches. This review will discuss the features of HLA-E, its immunological positioning, the potential impact of MS technologies in exploring the HLA-E immunopeptidome, and its role in developing universal anti-viral and anti-cancer immunotherapies that could serve as safe and effective alternatives to current treatment options.

## HLA-E expression patterns and stability in human tissues and cell types

**Figure 1:**
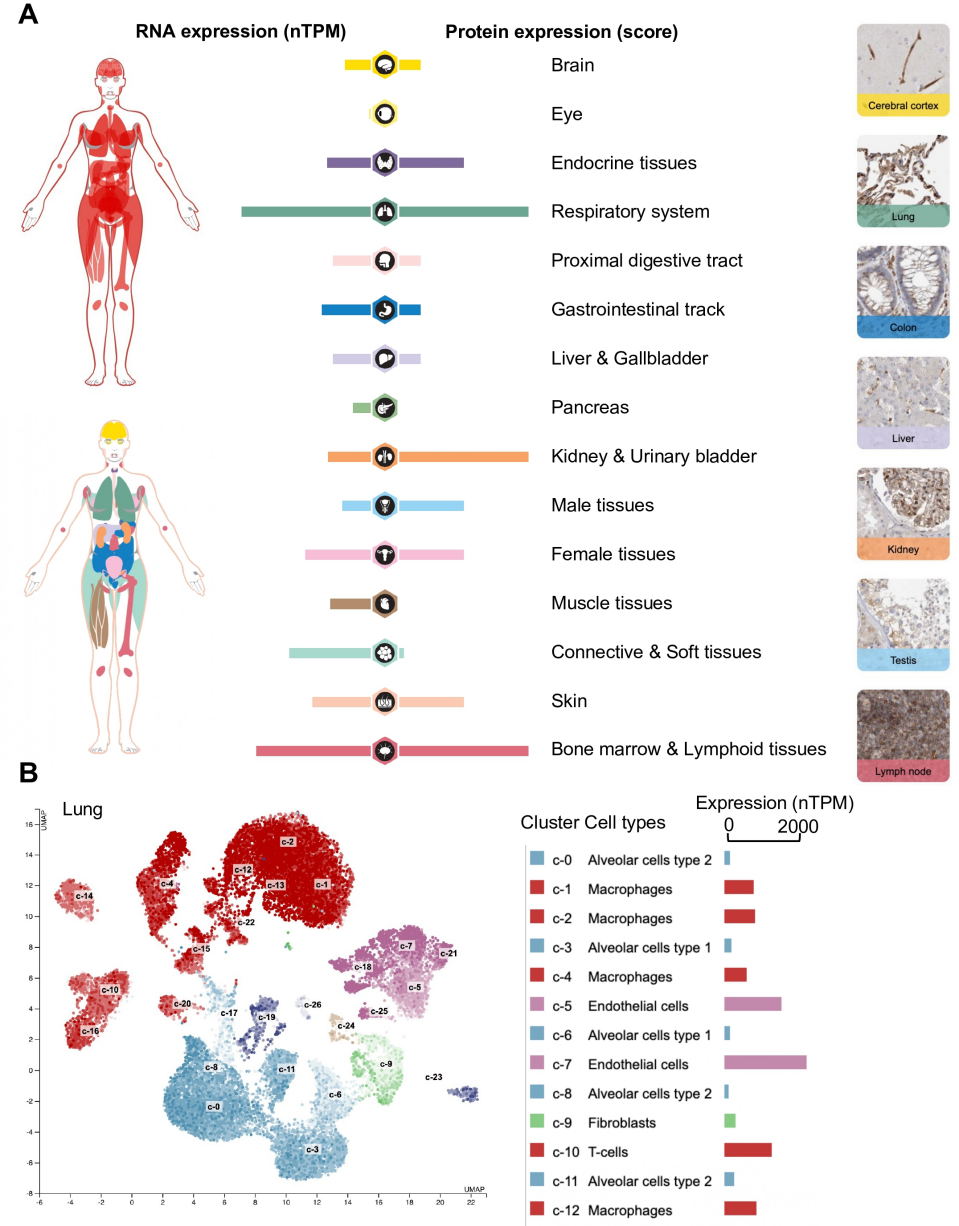
Exploration of HLA-E expression at the RNA and protein levels in human tissues and cell types using the Human Protein Atlas. **(A)** Expression profiles in human tissues of HLA-E both on the mRNA and protein level. The protein expression data from 44 normal human tissue types is derived from antibody-based protein profiling using conventional and multiplex immunohistochemistry. Each bar represents the highest expression score found in a particular group of tissues. Protein expression scores are based on a best estimate of the “true” protein expression from a knowledge-based annotation, described more in detail under Assays & annotation. The mRNA expression data is derived from RNA-seq from 40 different normal tissue types. RNA expression summary shows the consensus data based on normalized expression (nTPM) values from two different sources: internally generated Human Protein Atlas RNA-seq data and RNA-seq data from the Genotype-Tissue Expression (GTEx) project. **(B)** RNA expression of HLA-E at the single-cell level. The single-cell RNA sequencing (scRNAseq) data analysis was based on publicly available genome-wide expression data. Twelve out of twenty-six cluster cell types (right) are shown in the legend for simplicity. Use https://www.proteinatlas.org/ENSG00000204592-HLA-E for an in-depth exploration of HLA-E expression across human tissues and cell types. **(B)** was generated automatically using the Human Protein Atlas (https://www.proteinatlas.org/) (20).

Non-classical HLA-E has significantly lower RNA expression levels than classical HLA-A, -B, and -C. Specifically, a study showed that HLA-E transcripts exhibit the highest median expression in the blood (275 RPKM), spleen (187 RPKM), lung (142 RPKM), and adipose tissue (141 RPKM) whereas classical HLA-I molecules show the highest median expression levels in the whole blood (1210 RPKM), spleen (850 RPKM), bone marrow (615 RPKM), lung (433 RPKM), small intestine (482 RPKM), and lymph node (422 RPKM) (19). The Human Protein Atlas (https://www.proteinatlas.org/ENSG00000204592-HLA-E/tissue) is also a valuable resource for exploring HLA-E expression at both RNA and protein levels, providing bulk and spatial information of its expression across a wide range of tissues and cell types (**Figure 1**) (20). According to the Human Protein Atlas data, HLA-E expression appears to be ubiquitous yet predominant in the respiratory system, kidney, urinary bladder, bone marrow, and lymphoid tissues (**Figure 1A**). Upon examination at the single-cell level in the lung, endothelial cells exhibit the highest expression of HLA-E, followed by infiltrating T cells and macrophages (**Figure 1B**). Thus, expression data suggest that the HLA-E immunopeptidome is present across the entire organism (20).

Since >99% of the human population possesses either HLA-E*01:01 or HLA-E*01:03, HLA-E is relatively monomorphic. They differ by a single amino acid substitution: Arg (HLA-E*01:01) is replaced by Gly (HLA-E*01:03) at position 107, outside of the peptide-binding groove (21). The presence of Gly in HLA-E*01:03 provides increased thermal stability over Arg when bound to an equivalent peptide, resulting in a more stable expression of HLA-E*01:03 (15). Such increased stability potentially prolongs the interaction time between peptide-HLA-E (pHLA-E) complex and immune effector cells, which may generally improve the level of immunogenicity for antigenic HLA-bound peptides (22). Nevertheless, the stability of HLA-E molecules on cellular surfaces remains much lower compared to classical HLA-Ia molecules (23).

In this regard, thermostability (as a surrogate of stability) profiling of the immunopeptidome has recently been conducted for classical HLA-I-associated peptides (24). Still, it has rarely been performed for the HLA-E immunopeptidome. For instance, Walters et al. individually studied and compared the stability and structural features of the VL9- and non-VL9-HLA-E immunopeptides, demonstrating differences in peptide-binding motif and surface T cell recognition profiles (25). Thus, high-throughput profiling could provide valuable insights into predicting the duration of pHLA-E-TCR interactions, consequently improving the predictability of the likelihood of T-cell activation and peptide immunogenicity models.

Assessing the global thermostability of pHLA-E complexes could potentially yield crucial information for understanding diseases and developing universal T-cell-directed vaccines by shortlisting the potential targets. Key to this effort will be identifying the disease conditions that lead to the expansion of stable, disease-specific pHLA-E complexes recognized by CD8^+^ T cells. Indeed, the nomination of HLA-E-restricted T cell targets depends on establishing novel therapeutics by identifying new antigenic peptides that can stably bind to the HLA-E peptide clefts (pockets). This aim can be fulfilled with the tetramerization of peptide-HLA (pHLA) complexes to induce specific T cells. For example, Vaurs et al. produced photosensitive peptides to assemble HLA-E/pUV complexes and develop pHLA-E complexes through peptide exchange. They used ELISA to characterize the UV exchanges and utilized a new approach for peptide exchange detection by size exclusion chromatography (SEC) (26).

## What is the scope of peptides exclusively presented by HLA-E alleles: a narrow range or a broad spectrum? Insights from immunopeptidomics studies

For many years, it was believed that HLA-E primarily bound a limited set of highly conserved nonameric 9-mer self-peptides derived from the leader sequences of classical HLA-Ia proteins, characterized by the consensus sequence VMAPRTL/VV/L/FL, and hence termed VL9 peptides (27). However, recent discoveries have shown that HLA-E ligands are not restricted to VL9 peptides and can originate from multiple host proteins and pathogenic sources (**Table 1** and **Supplementary Table S1**). Additionally, various self-peptides presented by HLA-E molecules have been identified in MS-based immunopeptidomics studies (**Table 1** and **Supplementary Table S1**). For instance, in 2016, Celik et al. conducted an immunopeptidomic study using human HLA-negative B-lymphoblastoid cell lines (LCL.721.221 and LCL.721.220) transduced with lentiviral vectors encoding soluble forms of HLA-E*01:01 or HLA-E*01:03. They identified 56 peptides of varying lengths (9-17 amino acids) in the absence of class I leader peptides, originating from diverse cellular proteins including myosin-9, histones H1.5, H2A 1-D, H2B 1-J or 1-L, and several 60S ribosomal proteins (23). However, further experimental validation is needed to confirm the HLA-E specificity of the peptides identified by MS, as antibodies that may not be specific to HLA-E, such as W6/32 (anti-HLA-ABC), are often used (**Table 1**).

**Table 1:** A representative list of known HLA-E-associated epitopes originating from various protein sources †.

Peptide Sequence	Length	Material	Immunopeptidome Enrichment Method	MS Method	HLA-E peptide recognition by T-cell assay	Origin	Ref
VMAPRTLVL	9	Different cancer cell lines	N/A¶	N/A¶	NK−CTL or HLA-A2-CTL-based T cell assays	Human cytomegalovirus strains, UL40 protein	(28)
		Murine TAP2-deficient RMA-S/HLA-E cell line & Human fibroblast cell line HEK-293T	N/A¶	N/A¶	NK−CTL-based T cell assay		(29)
		HCMV-derived PBMCs	N/A¶	N/A¶			(30)
		CMV-derived PBMCs	N/A¶	N/A¶			(31)
		Chronic myelogenous leukemia K-562 cell line, transfected with HLA-E*01033	N/A¶	N/A¶			(32)
		Human B cell line; 721.221 cells	Immunoprecipitation by anti-HLA class-I-pan W6/32 mAb crosslinked to mouse IgG-sepharose beads to isolate HLA-E peptides	RP-LC (C18) coupled to Kratos Axima-CFR MALDI mass spectrometer	NK−CTL-based T cell assay	Human, ATP-binding cassette transporter multidrug resistance-associated protein	(33)
		LCL 721.221 derivative cells transfected with HLA-Cw0401, -Cw1502, -G1, -Gs, and LCL	Immunoprecipitation by anti-HLA class-I-pan W6/32 mAb crosslinked to protein A-Sepharose beads	Finnigan TSQ 7000 LC-MS/MS system	HLA-E peptide binding assay	HLA-E and AEH proteins, expressed in 721.221 cells	(34)
		-PBMCs derived from blood sample of a CMV-seropositive kidney-transplant patient -HLA-E-transfected (721.221-E) and un-transfected (721.221) B-EBV cell lines	N/A¶	N/A¶	-Antibody blocking and TCR-αβ/CD3/CD8 downregulation -^51^Cr Release Assay	-Human cytomegalovirus strains, UL40 protein -Recombinant protein facility of SFR26 (synthetic peptides)	(35)
VMAPRTLIL	9	UL40-transfected cell line 721.221	N/A¶	N/A¶	NK−CTL-based T cell assay	Human cytomegalovirus strains, glycoprotein UL40 protein	(36)
		HCMV-derived PBMCs					(30)
		CMV-derived PBMCs					(31)
		The murine TAP2-deficient T cell lymphoma and RMA-S, cell line cotransfected with human β2m and HLA-E*01033					(37)
		LCL 721.221 derivative cells transfected with HLA-Cw0401, -Cw1502, -G1, -Gs, and LCL	Immunoprecipitation by anti-HLA class-I-pan W6/32 mAb crosslinked to protein A-Sepharose beads	Finnigan TSQ 7000 LC-MS/MS system	HLA-E peptide binding assay	HLA-E and AEH proteins, expressed in 721.221 cells	(34)
VMAPRTLLL	9	Murine TAP2-deficient RMA-S/HLA-E cell line & Human fibroblast cell line HEK-293T	N/A¶	N/A¶	NK−CTL-based T cell assay	Human cytomegalovirus strains, UL40 protein	(29)
		CMV-derived PBMCs					(31)
		Murine TAP-2-deficient RMA-S cells					(38)
		LCL 721.221 derivative cells transfected with HLA-Cw0401, -Cw1502, -G1, -Gs, and LCL	Immunoprecipitation by anti-HLA class-I-pan W6/32 mAb crosslinked to protein A-Sepharose beads	Finnigan TSQ 7000 LC-MS/MS system	HLA-E peptide binding assay	HLA-E and AEH proteins, expressed in 721.221 cells	(34)
		-PBMCs derived from blood sample of a CMV-seropositive kidney-transplant patient -HLA-E-transfected (721.221-E) and un-transfected (721.221) B-EBV cell lines	N/A¶	N/A¶	-Antibody blocking and TCR-αβ/CD3/CD8 downregulation -^51^Cr Release Assay	-Human cytomegalovirus strains, UL40 protein -Recombinant protein facility of SFR26 (synthetic peptides)	(35)
SQAPLPCVL	9	Murine TAP-2-deficient RMA-S cells	N/A¶	N/A¶	NK−CTL-based T cell assay	Epstein Barr Virus, Lytic switch protein BZLF1	(38)
SQAQLPCLV	9						
VMAPRTLFL	9	Murine TAP-2-deficient RMA-S cells	N/A¶	N/A¶	NK−CTL-based T cell assay	Epstein Barr Virus, Lytic switch protein BZLF1	(38)
		LCL 721.221 derivative cells transfected with HLA-Cw0401, -Cw1502, -G1, -Gs, and LCL	Immunoprecipitation by anti-HLA class-I-pan W6/32 mAb crosslinked to protein A-Sepharose beads	Finnigan TSQ 7000 LC-MS/MS system	HLA-E peptide binding assay	HLA-E and AEH proteins, expressed in 721.221 cells	(34)
VTAPRTLLL	9	LCL 721.221 derivative cells transfected with HLA-Cw0401, -Cw1502, -G1, -Gs, and LCL	Immunoprecipitation by anti-HLA class-I-pan W6/32 mAb crosslinked to protein A-Sepharose beads	Finnigan TSQ 7000 LC-MS/MS system	HLA-E peptide binding assay	HLA-E and AEH proteins, expressed in 721.221 cells	(34)
VTAPRTVLL	9						
VMAPRTVLL	9						
YLLPRRGPRL (LLPRRGPRL*)	10	Patients with chronic hepatitis C	N/A¶	N/A¶	IFN-γ ELISPOT CD8^+^ T cell assay	Hepatitis C Virus, Peptide HCV core	(39)
PEIVIYDYM	9	-Cryopreserved human PBMCs -B-lymphoblastoid cell line 721.221 and murine RMA-S cells	N/A¶	N/A¶	Intracellular cytokine staining (ICS) assay	Human Immunodeficiency Virus	(40)
AISPRTLNA	9	-PBMCs derived from HIV-infected patients -HLA-E transfected K-562 cells	N/A¶	N/A¶	NK−CTL-based T cell assay	Human Immunodeficiency Virus Gag polyprotein	(41)
VMAPRALLL	9	PBMCs derived from donors	N/A¶	N/A¶	TAP-2^−/−^ murine T cell lymphoma RMA-S cell line	Human Leukocyte Antigen-Cw7 Leader Sequence	(42)
PELAKSAPAPK	11	HLA^−^/TPN^+/-^ LCL 721.221 and HLA^−^/TPN^+/-^ T2 cell lines transduced with sHLA-E*01:03	Large-scale soluble HLA technology to isolate HLA-E bound peptides, developed by (43)	nano-LC Ultra 2D HPLC system coupled to Orbitrap mass spectrometer	NK−CTL-based T cell assay	Human, 60 kDa heat shock protein, mitochondrial precursor	(23)
VGGTSDVEVNEK	12						
QMRPVSRVL	9	-K562 and 721.221 cell lines -Two human CD94/NKG2A+ (but killer Ig-like receptor [KIR]−) cytotoxic NK cell lines	N/A¶	N/A¶	NK−CTL-based T cell assay	Human, 60 kDa heat shock protein, mitochondrial precursor	(44)
SQQPYLQLQ	9	-Immature dendritic cells (iDCs) treated by a peptic-tryptic digest of gliadin and single-purified α-, β-, γ-, or ω-gliadin fractions -PBMCs and NK cells isolated from celiac patients or healthy donors	N/A¶	N/A¶	-IFN-γ-and TNF-α T cell ELISPOT assay -Gliadin-DC-NK assay	Human, Alpha/beta-gliadin A-I precursor	(45)
ALALVRMLI	9	Human B cell line; 721.221 cells	Immunoprecipitation by anti-HLA class-I-pan W6/32 mAb crosslinked to mouse IgG-Sepharose beads to isolate HLA-E peptides from lysate	RP-LC (C18) coupled to Kratos Axima-CFR MALDI mass spectrometer	NK−CTL-based T cell assay	Human, ATP-binding cassette transporter multidrug resistance-associated protein	(33)
GMKFDRGYI	9						
AMAPRTLLL	9						
RRYQKSTEL	9						
AAVLEYL	7	Synthesized peptides were loaded onto recombinant B-LCL 721.221 and TAP deficient T2 expressing HLA-E∗01:01	Large-scale affinity purification of sHLA-E molecules to isolate and identify sHLA-E-bound peptides (46)	nano-LC Ultra 2D HPLC coupled to an Orbitrap ion trap mass spectrometer	NK−CTL-based T cell assay	Human, Histone H2A type 2-B	(47)**
VMAPRTLFL	9						
SKGKIYPVGYY	11						
DVHDGKVVSTHEQ	13						
PKKTESHHKAKGK	13						
LGHPDTLNQGEFKEL	15						
LVDSGAQVSVVHPNL	15						
SLQGRTLIL	9	B6-CIITA-Ed cells infected either as mock or PR8	Immunoprecipitation by anti-MHC-II antibody (clone M5/114.5) cross-linked to Protein G Sepharose beads to isolate peptide-MHC-II complexes, described in (48)	UltiMate 3000 RSLCnano System (PepMap C18 column) coupled to an Orbitrap Fusion Lumos Tribrid™ mass spectrometer	IFN-γ enzyme-linked immunosorbent spot (ELISpot) assay	Influenza A	(49)
ASNENMETM	9						
IYSTVASSL	9						
TYQRTRALV	9						
MSLLGKTQIL	10						
MSLLERIPIL	10						
MYLLERIPIL	10						
VSLQERTQIL	10						
MSLQGRTLIL	10						
SSLENFRAYV	10						
RLPAKAPLL	9	-MHC-I null cell line K562 with Mamu-E*02:04 and HLA-E*01:03 transfectant -PBMCs derived from HIVGag and Mycobacteria tuberculosis (Mtb) vaccinated with the rhesus cytomegalovirus 68-1 strain [RhCMV68-1-HIVGag vector SIV vaccine where rhesus macaques stimulate Mamu-E-restricted responses to RL9HIV epitope].	N/A¶	N/A¶	Flow cytometric ICS assay used to measure CD8^+^ T cell responses (intracellular expression of IFNγ and TNFα) in mononuclear cell preparations from blood -HLA-E peptide binding affinity assay by micro-scale refold-sandwich ELISA method (50, 51)	Mycobacterium tuberculosis 44, Enoyl-[acyl-carrier-protein] reductase [NADH]	(52)
SMADRAENL	9	-PBMCs derived from healthy adults who produced or lacked IFNγ in response to Mtb-derived PPD -PBMCs from infants vaccinated with M. bovis Bacillus Calmette Guerin (BCG)	N/A¶	N/A¶	-IFNγ ELISA T cell assay -Single HLA-E expressing cell lines (K562 cells) were used to assess T cell responses to T cell lines, HLA-E transfectants loaded with peptides (labeled with 1 µCi ^51^Cr), and cytotoxic CD8^+^ T cell clones from PBMCs	Mtb HRv1286	(53)***
WMCDRAVDL						Mtb HRv2954c	
SMAGRAGQL						Mtb HRv3282	
EMVLRADQL						Mtb HRv0191	
DMLGRAGGL						Mtb HRv3015	
EMKTDAATL						Mtb HRv3874	
EMGRAPLDL						Mtb HRv2627c	
EMLTSRGLL						Mtb HRv1997	
GMGMVGTAL						Mtb HRv1737c	
PMADIAAAL						Mtb HRv1253	
GMQFDRGYL	9	-EBV-transformed lymphoblastoid B cell lines (B-LCL), blasts, and macrophages from PBMC derived from donors who took Ty21a vaccine -The HLA class I-deficient B cell line 721.221 and its transfectants (721.221.AEH/E6) transfected with HLA-E, expressing HLA-E*01:01	N/A¶	N/A¶	The human granzyme B IFN-γ ELISPOT assay	Salmonella enterica serovar typhi, Chaperonin GroEL	(54)
AMLQDIATL							
KMLRGVNVL							
VEGEALATL							
AAVEELKAL							
AVAKAGKPL							
KLQERVAKL							
552 HLA-E peptides (424 9-mer)	8-13	-HLA-I negative K562 cells transfected with HLA-E*01:03 -K562.HLA-E.B8 and K562.HLA-E.UL49.5 cells -Qa-1^b^ mouse homolog of HLA-E (expressing H2-D^b^/K^b^)	Affinity chromatography by antibody W6/32 cross-linked to the Sepharose beads followed by 10 kD filtration and RP-HPLC C18 fractionation	Thermo LTQ-FT Ultra mass spectrometer operated in data-dependent acquisition (DDA) mode	N/A	Non-endosomal sources	(55)
28 HLA-E peptides	7-11	-U373, DC, and A549 cells uninfected and infected with the H37Rv strain of Mtb -PBMCs collected apheresis from healthy, LTBI, and Mtb-infected adult donors	Affinity chromatography with VLDLr, W6/32 (anti-pan HLA class I), and 3D12 (anti-HLA-E) antibodies to isolate soluble HLA-E peptides followed by 2D-HPLC	Nano LC coupled to Sciex 5600 Triple TOF in DDA (for discovery) and SWATH (for quantification) modes	IFN-γ ELISPOT assay	13 Mtb source proteins (i.e., ftsH, iniB, lpqI, mpa, rplA, Rv0435c, Rv0634A, and Rv3479) and antigenic ESX operon proteins (e.g., EspA, ESAT-6, EsxG, EsxH and PE5.EsxG)	(56)
SEVENVSVNVHNPTG	15	-K562 cell line transfected with HLA-E*01:03 -NK cells cocultured with dendritic cells and loaded with the identified HLA-E peptides to detect NK responses	Immunopeptidome enrichment followed by size-exclusion chromatography to isolate peptide-HLA-E complexes followed by elution by mild acetic acid	Mass spectrometry in discovery mode (DDA) to identify HLA-E peptides	NK−CTL-based T cell assay	Human cytomegalovirus (HCMV) pp65-protein	(57)
TSGSDSDEELVTTER							
DSDEELVTTERKTPR							

†This table shows details on the material, T-cell assay for HLA-E epitope recognition, and immunoprecipitation methods used to isolate and purify HLA-E peptides, followed by MS-based immunopeptidomics techniques (in MS-based studies).

*This HCV-core 10-mer peptide might be bound to HLA-E as a 9-mer LLPRRGPRL peptide (58).

**Kraemer et al. studied 36 HLA-E peptides (7-16 mers), and we listed some examples.

***Joosten et al. studied 68 HLA-E peptides, and we listed the top 10 ones based on their HLA-E motif scores and binding affinity to the HLA-E allele calculated in this research paper. ¶ N/A: Not applicable.

**Figure 2:**
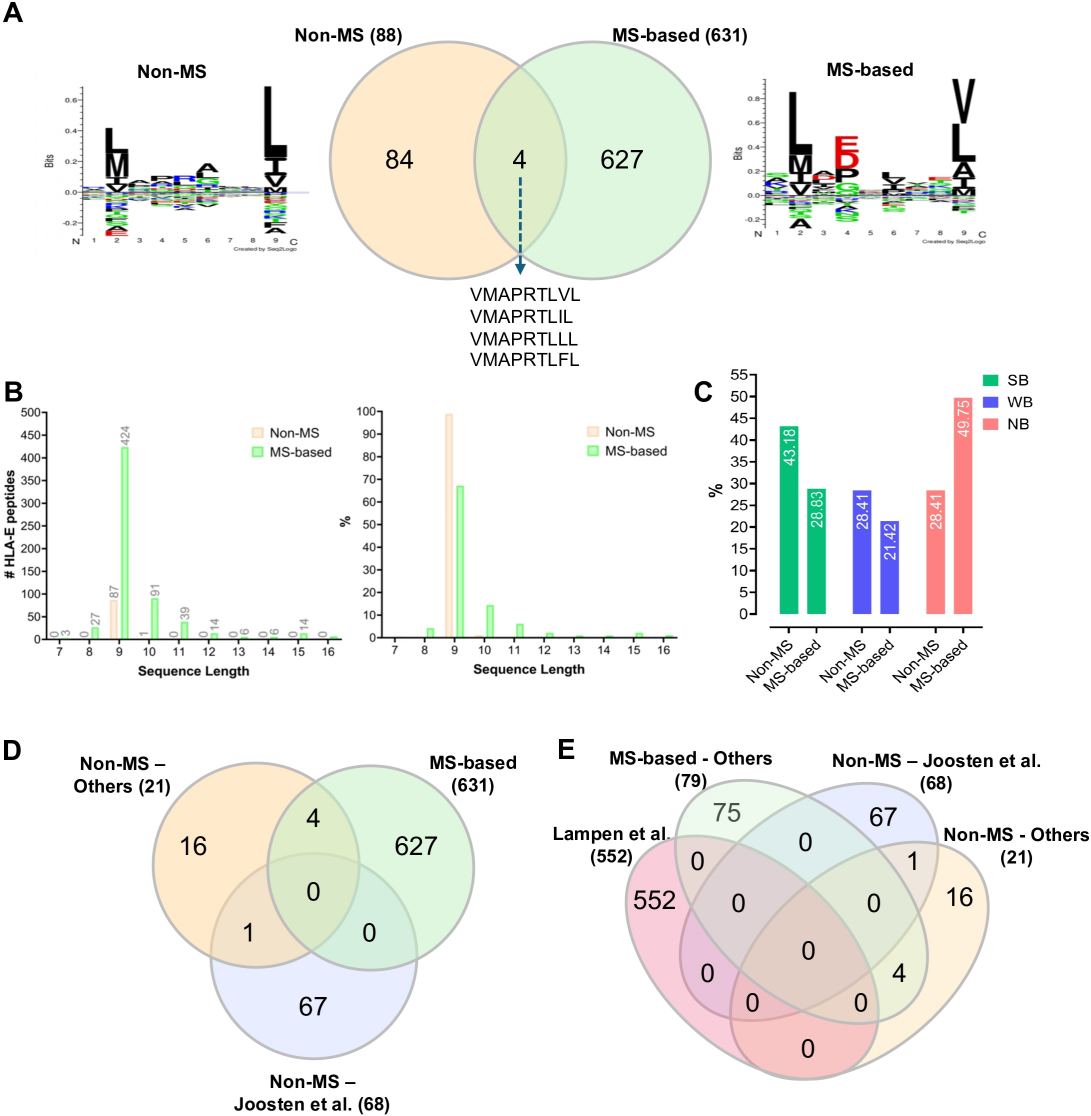
Comparative analysis of the previously reported HLA-E-bound peptides identified by non-MS *versus* MS-based immunopeptidomics techniques. **(A)** A two-way Venn diagram showing the overlap between the previously reported HLA-E-bound peptides and the corresponding sequence motif for each dataset. **(B)** Histogram showing length distribution of the previously reported HLA-E-bound peptides. **(C)** Histogram showing NetMHCpan 4.1 analysis of the previously reported HLA-E-bound peptides: percentages of predicted Strong Binders (SB); Weak Binders (WB) and Non-Binders (NB) are indicated (71). **(D)** A three-way Venn diagram comparing MS-identified HLA-E-bound peptides with findings from Joosten et al. (53), a notable non-MS study, and other non-MS studies. **(E)** A four-way Venn diagram illustrating the overlap among several studies that have characterized or examined higher numbers of HLA-E-bound peptides, including Lampen et al. (55) (MS-based), Joosten et al. (53) (non-MS), and other studies (see also **Supplementary Table S1**). Venn diagrams were generated by https://www.interactivenn.net/ (72). The sequence motifs were generated by Seq2Logo v. 2.0 (https://services.healthtech.dtu.dk/services/Seq2Logo-2.0/) (73) and GibbsCluster v. 2.0 (https://services.healthtech.dtu.dk/services/GibbsCluster-2.0/) (74) software tools. The bar graphs were generated by GraphPad Prism v. 9.3.1.

Proteasome-derived peptides must be bound to HLA class I molecules with enough binding affinity in the endoplasmic reticulum (ER) system to be presented on the cell surface for T cell immunosurveillance (59, 60). TAP (Transporter associated with Antigen Processing) is a key player in antigen processing, which transports peptide fragments from the cytosol into the ER system. Upon TAP impairment, new peptides have been reported to be presented by HLA-E. Specifically, in 2015, Kraemer et al. used TAP-deficient T2 cell lines transduced with soluble HLA-E*01:01. They found 36 HLA-E*01:01-bound peptides with 27 being longer than 10 amino acids (11-16 mers) and originating from various source proteins, including cell cycle regulatory proteins, matrix proteins, DNA repair proteins, and stress-induced proteins (47). However, those peptides need further experimental validation. In 2013, another study demonstrated that the repertoire of HLA-E-bound peptides retained the binding motif of HLA-A*02:01 upon TAP inhibition (55). Specifically, the authors used TAP-deficient K562 cells made by introducing the herpes virus evasion molecule UL49.5, which blocks TAP function and targets it for proteasomal degradation. Despite reduced peptide transport, surface HLA-E levels remained unchanged, indicating stabilization by an alternative, TAP-independent peptide repertoire (55). MS analysis of HLA-E-binding peptides from K562 cells revealed 552 unique peptide sequences in TAP-deficient cells (55) (**Supplementary Table S1**), with only eight overlapping with the TAP-proficient counterpart, indicating a distinct peptide repertoire in the absence of TAP function (55). Interestingly, the majority of the HLA-E peptide ligands were 9-mers, and few peptides were longer than 11 amino acids. The peptide sources were diverse, and many did not fit previously known processing mechanisms, suggesting other pathways, such as autophagy, might contribute to the TAP-independent peptide repertoire (55). Voogd et al. also described potential TAP-independent routes facilitating peptide processing and presentation onto HLA-E molecules (15). An alternative pathway involves furin, a protease in the trans-Golgi, which exhibits endoproteolytic activity by cleaving pro-proteins at designated C-terminal cleavage sites (15). Therefore, despite TAP impairment, HLA-E can present diverse arrays of peptides through alternative pathways. The lengths and identities of these peptides vary across different studies. In fact, a comprehensive analysis of HLA-E-bound peptides from multiple independent MS-based and non-MS studies reveals that these peptides are generally unique to each study, with minimal overlap (**Figure 2** and **Supplementary Table S1**). Four peptides were found to be common between MS and non-MS studies (**Figure 2A**). All peptides were 9-mers in non-MS studies whereas peptide lengths were more variable in MS-based studies (**Figure 2B**). Predicted binding affinity to HLA-E using NetMHCpan 4.1 indicates a ~2-fold increase in the proportion of non-binders (NB) in MS-based studies compared to non-MS studies (**Figure 2C**). These predictions should be approached with caution since prediction algorithms for HLA-E ligands are still relatively sub-optimal in comparison with classical HLA-ABC, and more data are required to improve their performances. Additional comparisons between several studies show limited overlap in common HLA-E peptides (**Figures 2D, E**). Thus, our analysis underscores the necessity for further investigations to enhance our understanding of the HLA-E immunopeptidome under various conditions, including TAP perturbations, viral infections, and oncogenic processes.

In this regard, tumor cells were shown to downregulate TAP subunits to avert T-cell clearance (61). In cancer, HLA-E peptides may take advantage of non-classical TAP-independent pathways if the classical ER-based, TAP-dependent route is unavailable. It has been found that murine TAP^-/-^ T-cell lymphoma cells present immunogenic alternative peptides on the mouse HLA-E ortholog, Qa1, and CD8^+^ T-cell clone production was restricted (15). These alternative peptides are unique and have little overlap with their TAP-proficient counterparts (55). One alternative peptide was discovered from MS-based immunopeptidomic procedures: a 9-mer epitope named M-SL9 (SLQGRTLIL) associated with influenza A virus (IAV) Puerto Rico/8/1934 (PR8) within IAV-infected C57BL/6 mice (**Table 1**). This epitope elicits a co-immunodominant, unconventional cytolytic T-cell response, uniquely presented by Qa-1–the murine homolog of HLA-E– with cryptic origin (49). Notably, it represents the first viral CD8^+^ T-cell epitope sequenced to be restricted to Qa-1 and does not interact with NK cells. Researchers originally intended to investigate cryptic epitopes presented on MHC-II following IAV infection. Through this procedure, they could detect the M-SL9 epitope from a cryptic viral translation product restricted to Qa-1 that drives a large percentage of the CD8^+^ T-cell response (49). The preliminary success of this procedure demonstrates the potential of identifying other viral cryptic antigens presented by HLA-E. On a technical note, this group employed an anti-MHC-II antibody that actually cross-reacted against Qa-1, leading to the unexpected isolation and detection of the cryptic epitope. With this notable cross-reactivity in mind, it has been found that antibodies 3D12 and 4D12 recognize HLA-E without cross-reacting with classical HLA-Ia molecules, serving as potential antibodies for future HLA-E-specialized immunopeptidomic procedures (62). However, it is worth mentioning that these two antibodies have different recognition patterns that could impact immunopeptidome recovery.

## Utilizing MHC-E-restricted CD8^+^ T cells in vaccines and immunotherapies against pathogens; viruses and cancer

MHC-E-restricted CD8^+^ T cells in T-cell-based vaccines and immune therapies offer distinct advantages over classically restricted CD8^+^ T cells. HLA-E molecules are ubiquitously expressed and have only two major allomorphs, which possess identical peptide-binding grooves. This characteristic enables the design of vaccines and therapies for the general population, eliminating the need for personalized approaches often required with MHC-Ia-restricted T cells (49). For instance, in the context of pathogen infections, a recent study described a TCR-based bispecific molecule that potently and selectively binds HLA-E in complex with a peptide encoded by the *inh*A gene of Mtb (63). The authors demonstrated the elimination of Mtb-infected cells and the reduction of intracellular Mtb growth. Moreover, the researchers revealed the biophysical and structural bases underpinning the potency and specificity of their TCR-based bispecific molecule, thereby providing a proof of principle for an innovative TCR-based therapeutic strategy capable of surmounting HLA polymorphism hurdles and thus promising broader applicability across patient populations.

Within the realm of vaccines, MHC-E-restricted CD8^+^ T cells have demonstrated distinctive protective abilities against HIV/SIV infections (64–66). HIV infection is generally known to downregulate classical HLA-Ia antigen expression via Nef proteins (67). In contrast, HLA-E expression is not affected by HIV infection and is continuously expressed to shield cells from NK-mediated lysis (68). The continuous expression of HLA-E upon infection has sparked interest in identifying and targeting MHC-E-associated epitopes for vaccine strategies against other viruses. To this end, an important aspect of consideration is the consistent and stable presentation of viral peptides within the HLA-E groove to ensure robust on-target T-cell activity and clinical effectiveness. High stability is particularly challenging for non-classical pHLA-E complexes, as they have been shown to be less stable than classical pHLA-Ia complexes (18). For instance, Wallace et al. recently employed immunopeptidomics in an effort to identify stable HLA-E-bound HIV-derived peptides that could be utilized to generate bispecific high-affinity T cell receptors (ImmTAX) (18). However, the instability of the predicted HIV-derived HLA peptide ligands posed a challenge in identifying HLA-E-bound peptides as potential therapeutic targets. As a result, Wallace et al. were unable to detect any HIV-derived HLA-E-bound peptides using MS-based immunopeptidomics techniques with the 3D12 antibody (18).

Recently, an mRNA vaccine was developed to trigger an unclassical CD8^+^ T-cell response against IAV within infected mice, demonstrating the feasibility of employing mRNA vaccines to investigate the regulative and protective functions of HLA-E-restricted CD8^+^ T cells (49). As aforementioned, we anticipate that systematic thermostability profiling studies of the HLA-E immunopeptidome after infection and/or vaccination will become critically important in the future to discover stable and immunogenic HLA-E peptides for the optimal design of universal HLA-E-restricted CD8^+^ T cell vaccines (24).

In the field of oncology, a recent study explored the potential of rhesus cytomegalovirus (RhCMV) vectors, genetically engineered to trigger an HLA-E-restricted CD8^+^ T cell response targeting tumor-associated antigens (TAAs), in generating an effective anti-tumor reaction against prostate cancer (69). Interestingly, the authors showed that their cancer vaccine could trigger an HLA-E-restricted CD8^+^ T cells response to non-canonical epitopes, including a peptide encoded by prostatic acidic phosphatase (PAP) (69). Their data indicate that T cell responses to TAAs were comparable to viral antigen-specific responses and, therefore, suggest that the HLA-E immunopeptidome can be exploited for CD8^+^ T cell-based immunotherapies against cancer.

Finally, the observed rapid internalization of HLA-E-peptide complexes into the cell could also represent an opportunity for therapeutic innovation. For instance, antibody-drug conjugates (ADCs) tailored to target precise TAAs bound to HLA-E could capitalize on rapid intracellular internalization, enabling highly effective delivery of potent cytotoxic agents directly into cancer cells, thereby acting as “biological missile” (70). The strategic screening of ADCs for optimal binding to HLA-E-TAA complexes and rapid internalization properties hold promise for significant therapeutic advancements in clinical applications.

## Conclusion

In summary, peptides presented by HLA-E, a non-classical HLA-Ib molecule, hold promise as actionable targets for a wide patient population. MS-based immunopeptidomics stands as a valuable tool in identifying these clinically relevant targets. Despite limited studies to date, employing MS-based immunopeptidomics has successfully uncovered both canonical and non-canonical CD8^+^ T cell epitopes presented by HLA-E molecules. As antibody specificity for isolation of pHLA-E and MS instrumentation sensitivity continue to improve, there is considerable potential for high-throughput discovery and validation of HLA-E viral epitopes and tumor antigens. In the context of anti-cancer immunotherapeutics, tumors often manipulate the antigen processing and presentation pathway to evade the immune response, such as through TAP-deficiency in various tumor cells. As discussed earlier, the downregulation of TAP leads to an expansion of the HLA-E immunopeptidome, suggesting a plausible avenue for anti-cancer treatments centered around targeting TAP-independent HLA-E epitopes. By continuing to explore and exploit the potential of the HLA-E immunopeptidome in immunotherapy using MS technologies, we may unlock new avenues for improving patient outcomes across a diverse range of diseases.
